# REDIdb 3.0: A Comprehensive Collection of RNA Editing Events in Plant Organellar Genomes

**DOI:** 10.3389/fpls.2018.00482

**Published:** 2018-04-11

**Authors:** Claudio Lo Giudice, Graziano Pesole, Ernesto Picardi

**Affiliations:** ^1^Institute of Biomembranes, Bioenergetics and Molecular Biotechnologies, Consiglio Nazionale delle Ricerche, Bari, Italy; ^2^Department of Biosciences, Biotechnology and Biopharmaceutics, University of Bari A. Moro, Bari, Italy

**Keywords:** organellar genomes, RNA editing, plant database, mitochondria, chloroplasts

## Abstract

RNA editing is an important epigenetic mechanism by which genome-encoded transcripts are modified by substitutions, insertions and/or deletions. It was first discovered in kinetoplastid protozoa followed by its reporting in a wide range of organisms. In plants, RNA editing occurs mostly by cytidine (C) to uridine (U) conversion in translated regions of organelle mRNAs and tends to modify affected codons restoring evolutionary conserved aminoacid residues. RNA editing has also been described in non-protein coding regions such as group II introns and structural RNAs. Despite its impact on organellar transcriptome and proteome complexity, current primary databases still do not provide a specific field for RNA editing events. To overcome these limitations, we developed REDIdb a specialized database for RNA editing modifications in plant organelles. Hereafter we describe its third release containing more than 26,000 events in a completely novel web interface to accommodate RNA editing in its genomics, biological and evolutionary context through whole genome maps and multiple sequence alignments. REDIdb is freely available at http://srv00.recas.ba.infn.it/redidb/index.html

## Introduction

RNA editing is an essential co/post transcriptional process able to expand transcriptome and proteome diversity in addition to alternative splicing. The term RNA editing was first introduced in 1986 to describe the addition and deletion of uridine nucleotides to and from mRNAs in trypanosome mitochondria (Benne et al., 1986). Since then, RNA editing events have been found in a wide range of organisms and can occur in the nucleus and cytoplasm as well as in organelles (Bowe and depamphilis, 1996). Modifications due to RNA editing comprise nucleotide substitutions and insertions or deletions that can affect both protein coding and Non-protein coding RNAs (Maier et al., 1996; Steinhauser et al., 1999).

In humans, the most prevalent type of RNA editing event is the deamination of adenosine (A) in inosine (I) in double RNA strands (dsRNAs) through the catalytic activity of the adenosine deaminase (ADAR) family of enzymes. To date, more than 4 million events have been collected and annotated in dedicated resources such as DARNED, RADAR, and REDIportal (Kiran et al., 2013; Ramaswami and Li, 2014; Picardi et al., 2017).

In plants, RNA editing occurs mostly in organelles in the form of cytidine (C) to uridine (U) conversion particularly in translated regions of mRNAs, albeit the opposite event (U-to-C substitutions) has been observed in some taxa, especially in chloroplasts RNAs (Takenaka et al., 2013). Plant RNA editing sites are recognized by specific pentatricopeptide repeat (PPR) proteins that are encoded in the nuclear genome. In flowering plants, the editosome machinery requires several additional Non-PPR protein factors, even though its molecular assembly has yet to be clarified (Sun et al., 2016).

Most of the C-to-U changes in the protein coding regions tends to modify affected codons restoring evolutionary conserved aminoacid residues (Gray, 2003). Therefore, plant RNA editing is believed to act as an additional proofreading mechanism to generate fully functional proteins. Occasionally, C-to-U modifications occur in untranslated regions, structural RNAs and intervening sequencing, affecting splicing and translation efficiency. Indeed, RNA editing changes in the domain V of plant group II introns is mandatory for the splicing process (Castandet et al., 2010).

With the advent of high-throughput sequencing technologies, many complete plant organellar genomes have been released and numerous novel RNA editing events uncovered. Nevertheless, RNA editing changes are not always correctly or completely annotated in primary databases (GenBank, ENA and DDBJ) and an appropriate field to unambiguously describe them is not provided. RNA editing modifications are often reported as misc_feature or even as simple exception notes. With the aim to overcome these limitations and create a cured catalog of plant RNA editing events, we developed the specialized REDIdb database. Its first release stored 9,964 modifications distributed over 706 different nucleotide sequences, increased to 11,897 in the following update.

After 10 years of massively parallel sequencing, we present here REDIdb 3.0, an upgraded release that annotates 26,618 RNA editing events distributed among 281 organisms and 85 complete organellar genomes.

All changes have been recovered from Genbank and literature using a semi-automated bioinformatics procedure in which each annotation has been manually checked to avoid redundancy or inconsistencies due to errors in flatfiles.

The web-interface was totally restyled and developed using the latest computational technologies in the field of database querying and managing.

Furthermore, many computational facilities have been integrated to improve the user experience and ensure continuous and future updates of the database. Indeed, REDIdb 3.0 accommodates RNA editing in its genomics, biological and evolutionary context through whole genome maps and multiple sequence alignments.

Although a variety of RNA editing databases have been released such as DARNED (Kiran et al., 2013), RADAR (Ramaswami and Li, 2014), and REDIportal (Picardi et al., 2017), REDIdb is the only one devoted to editing changes in plant organelles. Indeed, similar resources such as dbRES (He et al., 2007), RESOPS (Yura et al., 2009), ChloroplastDB (Cui et al., 2006), or GOBASE (O'Brien et al., 2009) have been dismissed or not updated.

## Materials and methods

All editing events stored in REDIdb derive from GenBank flatfiles through a semi-automated parsing algorithm implemented in custom python (2.7.13) scripts. Each flatfile is screened for RNA editing features using the SeqIO parser included in the Biopython (1.68) module (Cock et al., 2009).

All annotations have been manually checked to identify and correct potential errors, taking into account other related flatfile fields or literature. REDIdb database is organized in MySQL tables and queries are in python employing the MySQL-python (1.2.5) module, a data access library to MySQL engine. The web interface, instead, is built in BootStrap (3.3.7), while data presentation is based on DataTables, an *ad hoc* Javascript library (1.10.13) to efficiently show large tables in html documents. Genome rendering, available for complete organellar genomes, has been developed in pure python, mimicking OGDraw graphics (Lohse et al., 2013).

Query results are dynamically generated using the CGI (common gateway interface) technology. Multiple sequence alignments of edited cDNAs and proteins have been generated by ClustalOmega (Sievers et al., 2011) and displayed in html pages through the MSAViewer (Yachdav et al., 2016), a JavaScript component of the BioJS collection (https://biojs.net/).

The distribution of RNA editing events along functional domains and predicted protein secondary structures are shown by the feature-viewer JavaScript library (https://github.com/calipho-sib/feature-viewer) based on the powerful D3 JavaScript library for visualizing data using web standards (https://d3js.org/). Functional domains have been detected using InterPro engine (Jones et al., 2014), while protein secondary structures have been predicted using the stand-alone version of Spider2 program (Yang et al., 2017).

All the scripts to parse multiple alignments, InterPro html files and Spider2 outputs have been created in Python. Scripts used to extract RNA editing positions from Genbank flatfiles are freely available at the REDIdb help page. Additional details and supplementary scripts are available upon request.

## Results

### Database content

Previous REDIdb release contained 11,897 editing events distributed over 198 organisms and 929 different nucleotide sequences. This upgraded version, instead, collects more than 26,000 editing events from 281 organisms, 85 complete organellar genomes and 3,467 sequences. REDIdb 3.0 includes 26,545 events in protein coding sequences and 73 in untranslated regions, structural RNAs and introns. The vast majority of editing changes occur in the mitochondrion, accounting for a total of 23,553 events over 2,300 sequences.

The most recurrent RNA editing modification is the C-to-U substitution, that accounts for more than 92% of all annotated events and, when located in protein coding regions, tends to modify the aminoacid coded by the edited codon. Indeed, the majority of RNA editing events affects the first and second codon position leading to aminoacid changes resulting the most conserved in the comparison with related orthologs.

Differently from the previous releases, the novel REDIdb database annotates 85 complete organellar genomes. Of these 57 are mitochondrial genomes and include 7791 events. As reported in Table 1, the most edited mitochondrial genomes are those from *Liriodendron tulipifera, Nelumbo nucifera* and *Ginkgo biloba* with 888, 847, and 717 events, respectively. Of 27 annotated chloroplast genomes, instead, the one from *Anthoceros formosae* comprising 564 modifications results the richest in editing events.

**Table 1:** Number of RNA Editing events in complete genomes stored in REDIdb.

**Location**	**Protein_coding_events**	**No_protein_coding_events**	**Genbank_id**	**Organism_name**	**Cultivar**	**Strain**
mito	6		NC_021931	*Anomodon attenuatus*		
mito	6		NC_016121	*Anomodon rugelii*		
mito	493		NC_001284	*Arabidopsis thaliana*		
mito	2		NC_024520	*Atrichum angustatum*		
mito	1		NC_024519	*Bartramia pomiformis*		
mito	344		NC_015994	*Beta macrocarpa*		
mito	340		NC_015099	*Beta vulgaris* subsp. maritima		
mito	4		NC_031212	*Brachythecium rivulare*		
mito	417		AP006444	*Brassica napus*	Westar	
mito	467		KJ820683	*Brassica oleracea* var. botrytis		
mito		5	KJ820683	*Brassica oleracea* var. botrytis		
mito	2		NC_024518	*Buxbaumia aphylla*		
mito	2		NC_012116	*Carica papaya*	SunUp	
mito	547		NC_014043	*Citrullus lanatus*	Florida giant	
mito	3		NC_024515	*Climacium americanum*		
mito	475		NC_014050	*Cucurbita pepo*	Dark green zucchini	
mito	719		NC_027976	*Ginkgo biloba*		
mito		8	AY182006	*Harpochytrium* sp. JEL105		JEL105
mito		5	AY182005	*Harpochytrium* sp. JEL94		JEL94
mito	1		AP014526	*Hevea brasiliensis*	BPM 24	
mito	1		AP017300	*Hordeum vulgare* subsp. spontaneum		H602
mito	1		AP017301	*Hordeum vulgare* subsp. vulgare		HarunaNijo
mito	3		NC_026515	*Hyoscyamus niger*		
mito	5		NC_024516	*Hypnum imponens*		
mito	888		KC821969	*Liriodendron tulipifera*		
mito	488		NC_016743	*Lotus japonicus*		
mito		1	NC_016743	*Lotus japonicus*		
mito	488		JN872551	*Lotus japonicus*		MG-20
mito		1	JN872551	*Lotus japonicus*		MG-20
mito	485		NC_016742	*Millettia pinnata*		
mito		1	NC_016742	*Millettia pinnata*		
mito		10	AY182007	*Monoblepharella* sp. JEL15		JEL15
mito	847		NC_030753	*Nelumbo nucifera*		
mito	3		NC_029805	*Nicotiana sylvestris*	TW 137	
mito	1		NC_006581	*Nicotiana tabacum*	Bright Yellow 4	
mito	60		NC_012651	*Nothoceros aenigmaticus*		
mito		1	NC_012651	*Nothoceros aenigmaticus*		
mito	1		NC_029356	*Orthotrichum diaphanum*		
mito	1		NC_029355	*Orthotrichum macrocephalum*		
mito	2		NC_024522	*Orthotrichum stellatum*		
mito	3		AP017386	*Oryza sativa* Indica Group		BT-CMS
mito	97		NC_013765	*Phaeoceros laevis*		
mito	16		NC_017755	*Phlegmariurus squarrosus*		
mito	8		NC_013444	*Pleurozia purpurea*		
mito	8		NC_024514	*Ptychomnion cygnisetum*		
mito	60		JQ083668	*Raphanus sativus*		
mito	7		NC_027974	*Sanionia uncinata*		
mito	326		NC_014487	*Silene latifolia*		
mito	3		NC_024521	*Sphagnum palustre*		
mito	1		NC_017840	*Spirodela polyrhiza*		7498
mito	1		NC_027515	*Syntrichia filaris*		
mito	5		KC784953	*Tetraphis pellucida*		
mito	2		NC_028191	*Tetraplodon fuegianus*		
mito	1		NC_016122	*Treubia lacunosa*		
mito	2		NC_024517	*Ulota hutchinsiae*		
mito	7		NC_015121	*Vigna radiata*		
mito	109		AY506529	*Zea mays*		NB
chloro	79		KU764518	*Actinostachys pennula*		
chloro	343		AY178864	*Adiantum capillus-veneris*		
chloro		1	AY178864	*Adiantum capillus-veneris*		
chloro	564		NC_004543	*Anthoceros formosae*		
chloro	56		NC_019628	*Apopellia endiviifolia*		
chloro	25		LC154068	*Arabidopsis lyrata* subsp. lyrata		MN47
chloro	37		NC_000932	*Arabidopsis thaliana*		
chloro	36		NC_004561	*Atropa belladonna*		Ab5p(kan)
chloro	1		NC_031894	*Citrus depressa*		
chloro	1		NC_009618	*Cycas taitungensis*		
chloro	65		NC_028542	*Cyrtomium devexiscapulae*		
chloro	55		NC_028705	*Cyrtomium falcatum*		
chloro	2		NC_031159	*Ipomoea nil*	Tokyo-kokei standard	
chloro	1		NC_016058	*Larix decidua*		
chloro	3		NC_017006	*Mankyua chejuensis*		
chloro	38		NC_016708	*Millettia pinnata*		
chloro		1	NC_016708	*Millettia pinnata*		
chloro	58		NC_014592	*Myriopteris lindheimeri*		
chloro	40		NC_001879	*Nicotiana tabacum*	Bright Yellow 4	
chloro	5		NC_007602	*Nicotiana tomentosiformis*		
chloro	44		AY916449	*Phalaenopsis aphrodite* subsp. *formosana*	Taisugar TS-97	
chloro	1		NC_005087	*Physcomitrella patens* subsp. patens		
chloro	23		NC_001631	*Pinus thunbergii*		
chloro	66		NC_014348	*Pteridium aquilinum* subsp. aquilinum		
chloro	16		NC_005878	Saccharum hybrid cultivar SP80-3280		
chloro	1		NC_020098	*Tectona grandis*		
chloro	72		NC_028543	*Woodwardia unigemmata*		
chloro	32		NC_001666	*Zea mays*		

*Events are divided by sequence (coding/Non-coding) and according to their intracellular location. In presence of multiple accession numbers for the same organism, only the RefSeq record (if present in Genbank) has been considered*.

All REDIdb sequences including RNA editing events are identified by unique accession numbers (e.g., EDI0000.). To preserve the full compatibility with previous database versions, accession numbers linked to old entries have been maintained unchanged.

### Query form and output tables

**Figure 1:**
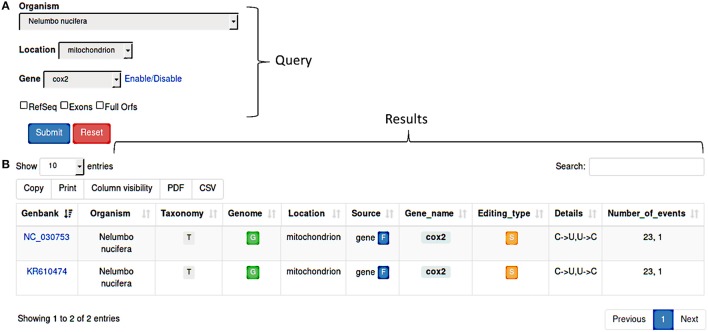
**(A)** REDIdb query form. Searches can be performed by Organism, Location, Gene or a combination of them. Additional filters (RefSeq, Exons, Full Orfs) are also available in order to refine the results. **(B)** Once a query has been submitted, the corresponding results are displayed in a sortable and exportable table report.

REDIdb implements a modular query form (Figure 1A) allowing users to make flexible searches by selecting the organism or the intracellular location or the gene name. Regarding nucleotide sequences, users can retrieve the original sequence submitted to the primary database or the RefSeq version or both. In addition, the search can be limited to full open reading frames and include individual exons in case of interrupted genes.

Query results are shown in a sortable and exportable summary table (Figure 1B) comprising several info such as the GenBank accession number, the organism and the link to the related taxonomy, the organelle type and the link to the complete genome (if available), the gene name and a flag indicating its partial or full nature, the editing types and details and the total number of events. Column can be selectively included in the final table and results are downloadable in pdf or csv format. The “Taxonomy” column includes a link to an interactive taxonomy chart, while the “Genome” column contains a link to the complete genome (if available in primary databases) chart in which RNA editing events are displayed in their genomics context.

Using the link in the “Gene_name” column, users can browse individual RNA editing events organized in flatfiles.

### Entry organization

**Figure 2:**
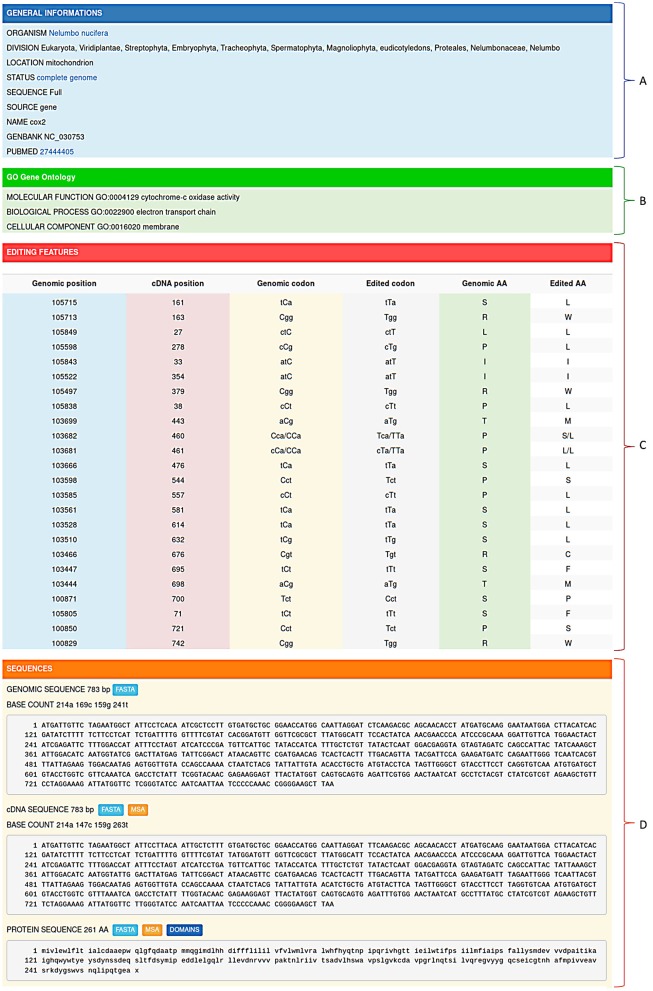
Editing informations stored in REDIdb are organized in specific flat-files in which it is possible to distinguish a header **(A)** containing the main features of the record (organism, Genbank accession, intracellular location, gene name, PubMed references, ecc.), a gene ontology box **(B)** describing the gene product properties, a feature table **(C)** with all the editing events and a sequence zone **(D)** with both the genomic sequence and the corresponding edited transcript/protein.

RNA editing events stored in REDIdb are organized in specific flat-files comprising four main sections. The first section (Figure 2A) contains a general description of the entry including the organism name, the taxonomy (according with the NCBI Taxonomy database), the GenBank and PubMed accession numbers, the intracellular location (mitochondrion or chloroplast) and the official gene name.

The second section (Figure 2B) is devoted to Gene Ontologies (GO), obtained by matching each protein sequence contained in REDIdb against the InterPro database (Finn et al., 2017). In the case of protein coding genes, it contains information regarding the molecular functions, the biological processes and the cellular localization of the protein product. The third section (Figure 2C) shows all the editing features that characterize the record. Here, for each editing event the position on the transcript is reported and, if the complete reference genome is available, also the genomic location. In case of editing within protein coding genes, the genomic codon, edited codon and aminoacidic change are determined and reported. Finally, the fourth section (Figure 2D) contains the genomic sequence and the corresponding edited transcript. In coding protein genes, also the edited protein is displayed. Genomic sequences as well as edited transcripts and proteins can be retrieved in Fasta format.

### Graphical visualization

**Figure 3:**
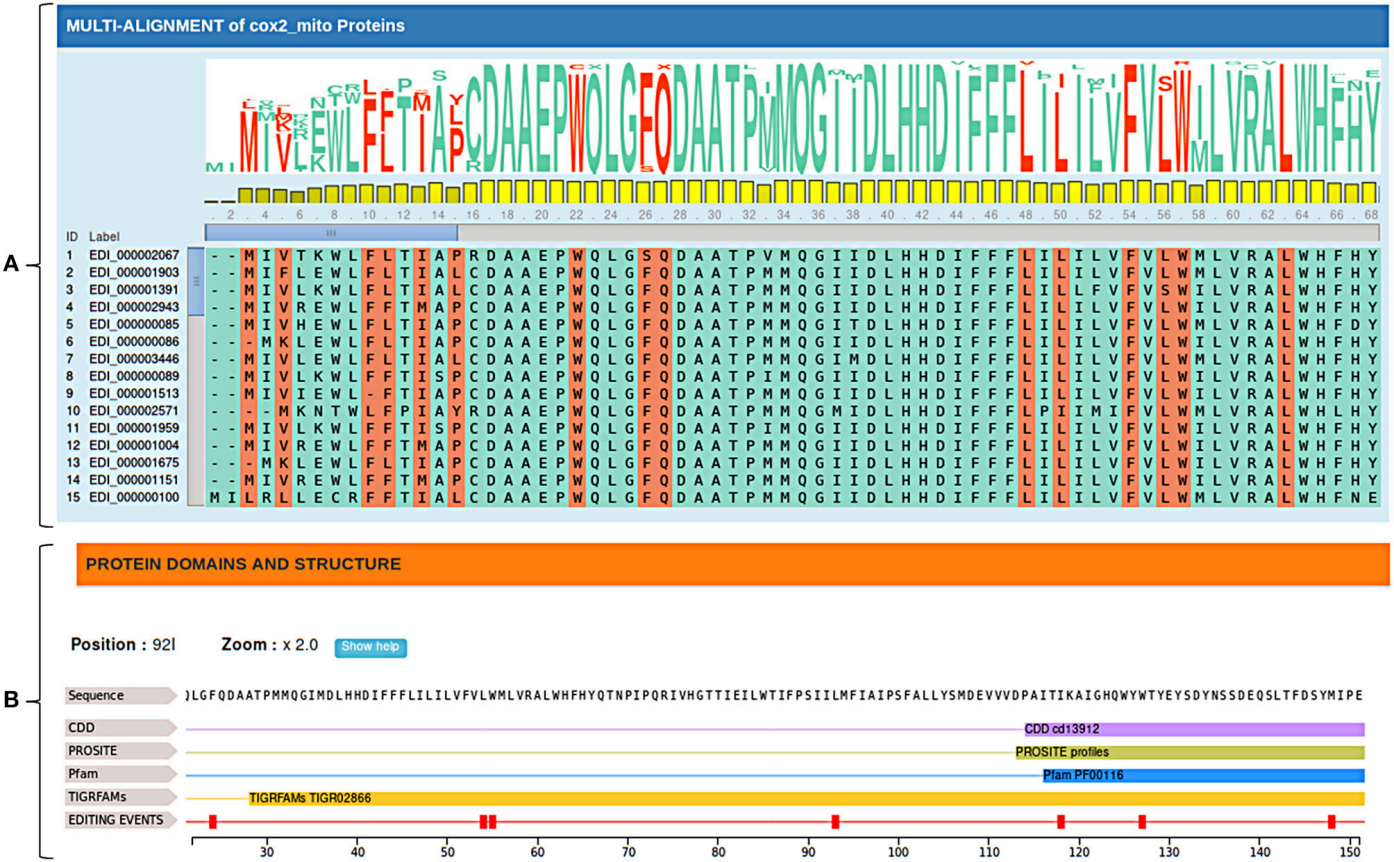
**(A)** Multiple alignments of cox2 orthologous sequences. Editing conservation across species can be easily obtained considering both the sequence logo and the bar chart relative to each position. **(B)** Protein domains and structure of *Nelumbo nucifera*'s cox2 protein. Protein domains are obtained by querying multiple databases (CDD, PROSITE, Pfam ecc.).

Edited cDNA and protein sequences can be explored in their evolutionary context through multiple alignments of available orthologs sequences. Since plant RNA editing tends to increase the sequence conservation along the evolution, annotated RNA editing changes are marked and visualized in the multiple alignment by the MSAViewer, to give rise to conservation levels and provide valuable comparative genomics information (Figure 3A).

In addition, RNA editing events are displayed along the edited sequence showing known functional domains and predicted secondary protein structures in order to better interpret the biological role of specific C-to-U or U-to-C changes (Figure 3B).

**Figure 4:**
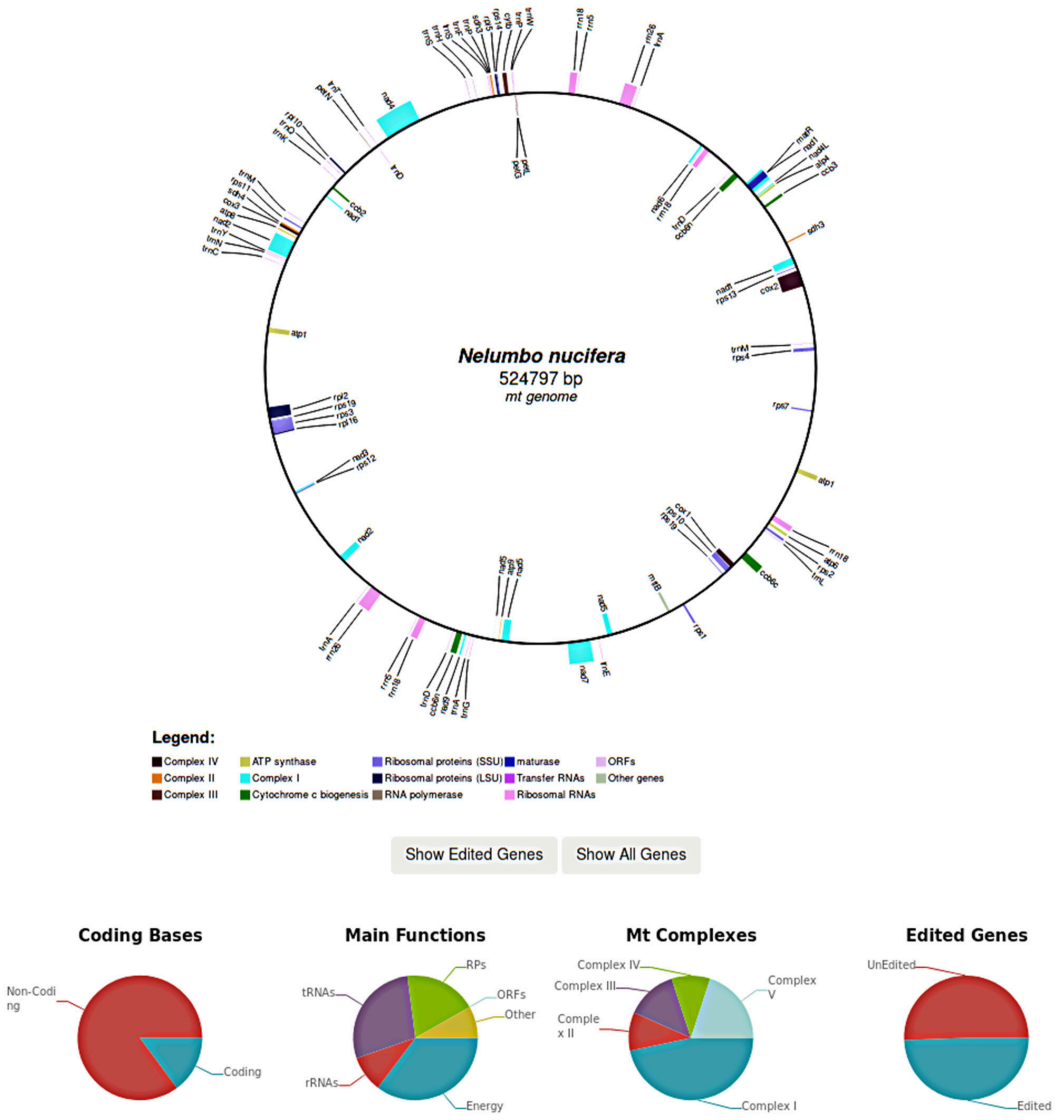
In REDIdb 3.0 complete genomes are graphically rendered, allowing users to visualize the reciprocal order of the edited genes. Further statistics such as the coding potential of the genome as well as the fraction of edited genes are reported in the same page.

In case of complete organellar genomes, each genome is graphically rendered and edited genes can be selectively highlighted. Genome graphs are generated in SVG and include links to edited genes by mousing over. Further statistics such as the coding potential of the genome as well as the fraction of edited genes are also reported (Figure 4).

## Conclusions and perspectives

As already mentioned, RNA editing plays an important role in transcriptome and proteome diversity. Since its first discovery in 1986 (Benne et al., 1986), a large number of events have been found in a wide range of eukaryotic organisms (Ichinose and Sugita, 2016). Only in humans more than 4 million events have been reported and dedicated resources such as DARNED, RADAR, and REDIportal have been developed to contain them into suitable specialized databases (Kiran et al., 2013; Ramaswami and Li, 2014; Picardi et al., 2017).

In the plant kingdom, RNA editing was first identified as C-to-U substitutions in mitochondrial transcripts (Hiesel et al., 1989), followed by its identification also in chloroplasts (Höch et al., 1991). In order to maintain a cured catalog of such events, we developed the specialized REDIdb database. Its third release, described here, contains three times more entries than the first version and two times more entries than the second version. To date, REDIdb is the unique bioinformatics resource collecting plant organellar RNA editing events. Indeed, similar databases such as dbRES (He et al., 2007) or RESOPS (Yura et al., 2009) have been dismissed or are no more updated. Plant RNA editing events are also annotated in CloroplastDB (Cui et al., 2006), devoted to chloroplast genomes, and GOBASE (O'Brien et al., 2009), the organelle genome database. However, such resources are not specialized for RNA editing and include potential not fixed errors due to the lack of manual curation (Picardi et al., 2011).

REDIdb 3.0 has been completely redrawn keeping in mind the simplicity as its working principle. RNA editing events are always shown in their biological context and novel graphical facilities have been added. Edited genes are now depicted in complete genome maps and RNA editing conservation can be investigated in pre-calculated multiple alignments of orthologous sequences. REDIdb 3.0 allows also the visualization of aminoacid changes induced by RNA editing in protein domains or secondary structures, providing insights into the potential functional consequences.

Next generation sequencing technologies, now arrived at their third generation, are expected to greatly increase the number of RNA editing candidates in the next future. Therefore, it will be indispensable to collect and annotate them in their biological context taking into account also the RNA editing levels.

Due to the unicity in its field, REDIdb is planned to be maintained and updated over time (as new editing sites or complete genomes are released), taking into account, as much as possible, eventual feedbacks from the users.

## Author contributions

CL conducted the bioinformatics analyses and wrote the first manuscript draft; EP and GP conceived the study and contributed to writing and revising the manuscript.

### Conflict of interest statement

The authors declare that the research was conducted in the absence of any commercial or financial relationships that could be construed as a potential conflict of interest.
